# Neue Techniken zur Quantifizierung des Farbsinns bei Störungen der Zapfenfunktion: Cambridge-Color-Test und photorezeptorspezifische zeitliche Kontrastempfindlichkeit bei Patient/Innen mit heterozygoten *RP1L1*- und *RPGR*-Mutationen

**DOI:** 10.1007/s00347-020-01119-0

**Published:** 2020-05-26

**Authors:** Cord Huchzermeyer, Julien Fars, Heidi Stöhr, Jan Kremers

**Affiliations:** 1 Augenklinik mit Poliklinik, Universitätsklinik Erlangen, Erlangen, Deutschland; 2 Institut für Humangenetik, Universität Regensburg, Regensburg, Deutschland

**Keywords:** Zapfendystrophie, Retinitis pigmentosa, Farbsehen, Photorezeptoren, Kontrastempfindlichkeit, Cone dystrophy, Retinitis pigmentosa, Color vision, Photoreceptors, Contrast sensitivity

## Abstract

**Hintergrund:**

Erbliche Netzhauterkrankungen mit Zapfendysfunktion können trotz relativ unauffälligem Fundusbefund ausgeprägte Visusminderung und deutliche Farbsinnstörungen aufweisen. Beispiele hierfür sind die autosomal-dominante okkulte Makuladystrophie (*RP1L1*-Gen) und die X‑chromosomale Retinitis pigmentosa (*RPGR*-Gen) – Letztere auch bei heterozygoten, weiblichen Merkmalsträgerinnen (Konduktorinnen). Neue Untersuchungsmethoden erlauben es, das Ausmaß der Farbsinnstörung zu quantifizieren.

**Methoden:**

Nach einer umfangreichen klinischen Untersuchung führten wir Messungen zur Quantifizierung der Farbdiskriminierung und der Zapfenfunktion durch. Beim Cambridge-Color-Test werden pseudoisochromatische Tafeln mit Landolt-C-Figuren computergesteuert generiert, um die Farbunterscheidungsschwelle entlang mehrerer Achsen im Farbraum zu bestimmen. Bei der Untersuchung der photorezeptorspezifischen zeitlichen Kontrastempfindlichkeit kann durch geschickte zyklische Veränderung der spektralen Zusammensetzung eines Lichtreizes die Kontrastwahrnehmungsschwelle isolierter Photorezeptortypen bestimmt werden. Die molekulargenetische Diagnostik erfolgte mithilfe von *Next Generation Sequencing*(NGS)-basierter gezielter Genpanelanalyse sowie Sanger-Sequenzierung.

**Ergebnisse:**

Bei 2 Patienten mit okkulter Makuladystrophie und 2 heterozygoten Trägerinnen von *RPGR*-Mutationen zeigten sich eine deutlich verminderte Fähigkeit zur Farbdiskriminierung und eine verminderte photorezeptorspezifische zeitliche Kontrastempfindlichkeit.

**Diskussion:**

Bei erblichen Netzhauterkrankungen sind neben den modernen bildgebenden Verfahren (okuläre Kohärenztomographie [OCT] und Fundusautofluoreszenz) auch die sinnesphysiologischen Untersuchungen diagnostisch wegweisend – der Nachweis von Farbsinnstörungen spielt hierbei eine wichtige Rolle. Neuere Methoden erlauben eine Quantifizierung der Farbsinnstörungen und könnten in klinischen Studien zu gen- und stammzellbasierter Therapie zur Messung des Therapieerfolges dienen.

## Abstract (en)

**Background:**

Inherited retinal diseases with cone dysfunction can be accompanied by severe visual loss and a marked loss of color vision despite relatively normal fundus appearance. Autosomal dominant occult macular dystrophy (*RP1L1* gene) and X‑chromosomal retinitis pigmentosa (*RPGR* gene, including heterozygous female carriers) are important examples. New examination techniques enable quantification of the extent of color vision disturbances.

**Methods:**

After a thorough clinical examination, color discrimination and cone function were quantified. The Cambridge color test is a computer-based test that generates pseudo-isochromatic plates with Landolt C figures for quantifying color discrimination along several axes in color space. Examination of photorecepor-specific temporal contrast sensitivity is performed by subtle cyclic modulation of the spectral composition of a light stimulus. Molecular diagnostics were carried out by next generation sequencing (NGS)-based targeted gene panel analysis and Sanger sequencing.

**Results:**

Markedly reduced color discrimination as well as reduced photoreceptor-specific temporal contrast sensitivity could be demonstrated in two patients with occult macular dystrophy and two heterozygous female carriers of *RPGR* mutations.

**Conclusion:**

The demonstration of dyschromatopsia is very helpful in the diagnosis of inherited retinal diseases, in addition to modern imaging techniques, such as optical coherence tomography (OCT) and fundus fluorescence. New functional techniques enable quantification of color vision disturbances and could be useful as outcome parameters in clinical trials of new gene and stem cell-based therapies.

Erbliche Netzhauterkrankungen mit Zapfenbeteiligung gehen in der Regel mit deutlichen Farbsinnstörungen einher. Dies gilt z. B. für die okkulte Makuladystrophie (*RP1-like Protein 1*-Gen, Chromosom 8), aber auch für die X‑chromosomale *RPGR*-assoziierte Retinitis pigmentosa (RP), sogar bei heterozygoten, weiblichen Merkmalsträgerinnen. Bei (relativ) unauffälligem fundoskopischem Befund können eine ausgeprägte Visusminderung und eine deutliche erworbene Farbsinnstörung bestehen. Letztere wird von den Patienten selten aktiv bemerkt – sie lässt sich nur durch geeignete Tests feststellen.

## Hintergrund

**Figure 1:**
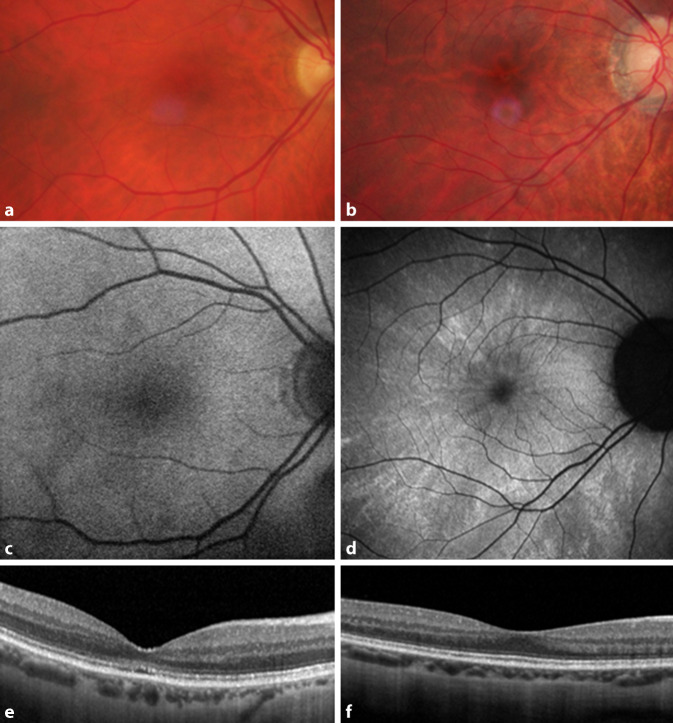

Netzhautdystrophien mit Dysfunktion der Zapfen können eine diagnostische Herausforderung darstellen, da sie häufig trotz deutlicher Visusminderung einen relativ unauffälligen Fundusbefund aufweisen [1, 27]. Dies zeigt sich auch in Abb. 1. Neben den modernen bildgebenden Verfahren (okuläre Kohärenztomographie [14] und Fundusautofluoreszenz [27]) können hier sinnesphysiologische Untersuchungen einen diagnostischen Beitrag leisten (Psychophysik [3, 14] und Elektrophysiologie [1]). Aufgrund der Variabilität des klinischen Phänotyps wird eine molekulargenetische Bestätigung der klinischen Diagnose heutzutage empfohlen.

Mutationen im *RP1L1*-Gen sind verantwortlich für die autosomal-dominant vererbte okkulte Makuladystrophie (OMIM #613587) [1, 20]. Bei dieser Erkrankung kommt es zu einem progredienten, auf den Bereich der Makula begrenzten Zapfenverlust [1]. Fundusbefund und Fluoreszenzangiographie sind typischerweise unauffällig, sodass die Diagnose in der Vergangenheit auf einer sorgfältigen klinischen Untersuchung und einem multifokalen ERG beruhte [1, 20]. Durch die weite Verbreitung der hochauflösenden okulären Kohärenztomographie (OCT) wird die Diagnose erleichtert, da sich hier Unregelmäßigkeiten in der ellipsoiden Zone (Übergang von den Innen- zu den Außensegmenten der Photorezeptoren) sowie verkürzte Photorezeptoraußensegmente beobachten lassen [4, 14]. Der Visus fällt üblicherweise nicht unter 0,1 bis 0,2 [1].

Im Gegensatz dazu werden Mutationen im *RPGR*-Gen mit der X‑chromosomalen Retinitis pigmentosa in Verbindung gebracht [16]. Abhängig von der Mutation zeigt sich jedoch auch eine deutliche Beteiligung der Zapfen mit frühem Verlust der zentralen Sehschärfe [18]. Eine diagnostische Herausforderung können bei dieser Erkrankung die heterozygoten weiblichen „Konduktorinnen“ darstellen, welche in unterschiedlichem Ausmaß auch von Symptomen betroffen sind [17]. Bei diesen Patientinnen zeigt sich im OCT oft ein regelrechtes Bild, in der Fundusautofluoreszenz hingegen ein pathognomonisches Muster [27]. Eine Störung der Zapfenfunktion ist bei diesen Patientinnen ebenfalls maßgeblich für die Symptomatik verantwortlich.

Die strikte Unterscheidung in eine Makuladystrophie auf der einen Seite und eine Retinitis pigmentosa auf der anderen Seite wird der Realität jedoch nicht gerecht. So sind auf der einen Seite auch *RP1L1*-assozierte Fälle von Retinitis pigmentosa [5], auf der anderen Seite auch Zapfen-(Stäbchen‑)Dystrophien bei *RPGR*-Mutationen beschrieben [6]. Dies spiegelt die Breite und Heterogenität der klassischen Diagnosekategorien wider. Auf der anderen Seite ist es auch die Rechtfertigung, im Rahmen dieser Studie beide Krankheitsbilder einander gegenüberzustellen und den Einfluss auf den Farbsinn zu vergleichen.

Das sinnesphysiologische Labor in Erlangen befasst sich mit innovativen Untersuchungstechniken des Farbsinns. Neben ihrer diagnostischen Wertigkeit haben solche sinnesphysiologische Untersuchungen auch das Potenzial, im Rahmen von klinischen Erprobungen neuer gen- oder zellbasierter neuroprotektiver Therapieansätze eine Rolle als funktioneller Endpunkt zu spielen. Im Rahmen des Schwerpunktprogramms 2127 („Gen- und Zellbasierte Therapien für die Behandlung neuroretinaler Degeneration“) der Deutschen Forschungsgemeinschaft untersuchen wir potenzielle Einsatzmöglichkeiten solcher innovativen Verfahren in klinischen Studien.

## Ziel der Arbeit

Es sollen Möglichkeiten dargestellt werden, Störungen der Zapfenfunktion mithilfe neuer Verfahren zu quantifizieren. Wir berichten exemplarisch von 2 Patienten mit *RP1L1*-assoziierter okkulter Makuladystrophie und von 2 Konduktorinnen mit heterozygoten *RPGR*-Mutationen.

## Methoden

Es erfolgte eine umfassende klinische Untersuchung mit bestkorrigiertem Visus, Perimetrie (Octopus 900, Haag-Streit, Köniz, Schweiz), Spaltlampenuntersuchung, Fundoskopie in Mydriasis, Spectral-Domain-OCT (HRA, Heidelberg Engineering, Heidelberg, Deutschland), Fundusautofluoreszenz und Elektroretinogramm (Retiport, Roland Consult, Brandenburg a. d. Havel, Deutschland). Der Farbsinn wurde mittels HCM-Anomaloskop (Oculus, Wetzlar, Deutschland) und dem Cambridge-Color-Test (Metropsis, Cambridge Research Systems, Rochester, England) untersucht. Außerdem erfolgte die Messung der zeitlichen Kontrastempfindlichkeit auf photorezeptorisolierende Reize (L-, M‑, S‑Zapfen und Stäbchen) mittels eines speziellen Leuchtdiodenstimulators. In allen 4 Fällen wurde die klinische Diagnose mittels einer NGS-basierten Genpanelanalyse molekulargenetisch gesichert.

### Cambridge-Color-Test

**Figure 2:**
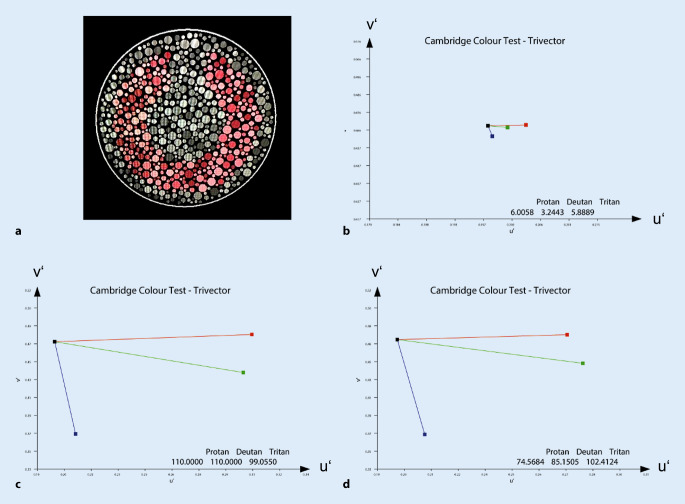

Der Cambridge-Color-Test untersucht die Farbdiskriminierungsschwelle computerbasiert mittels pseudoisochromatischer Tafeln, welche randomisiert und an die Antworten der Versuchsperson angepasst dargeboten werden [19, 23]. Der Reiz besteht (ähnlich wie bei den Ishihara-Tafeln) aus multiplen Farbflecken, welche unterschiedliche Größe, Helligkeit, Sättigung und Farbe aufweisen. Nur die farblichen Unterschiede zwischen den Flecken sind nicht zufällig und lassen die Versuchsperson ein Landolt‑C erkennen (Abb. 2). Die Richtung der Öffnung (oben, unten, links, rechts) wird durch Tastendruck rückgemeldet. Zur Messung verwendeten wir das Metropsis-System der Firma Cambridge Research Systems (CRS, England). Die Untersuchung wurde in einem abgedunkelten Raum in 4 m Abstand vom Bildschirm dargestellt, wobei die Öffnung des C’s in einem Winkel von 1° erscheint.

Es gibt verschiedene Messprogramme, welche das Farbunterscheidungsvermögen entlang der 3 Hauptachsen des CIE-u’v’-Farbraumes beim von uns verwendeten Trivector-Test (Protan‑, Deutan- und Tritan-Achse, Dauer 3–4 min) oder entlang mehrerer Achsen (Ellipse, Dauer ~25 min) messen.

### Zeitliche Kontrastempfindlichkeit der Fotorezeptoren

Die zeitliche Kontrastempfindlichkeit wurde mit einem eigens dafür hergestellten Leuchtdiodenstimulator [22] in einem abgedunkelten Raum untersucht.

**Figure 3:**
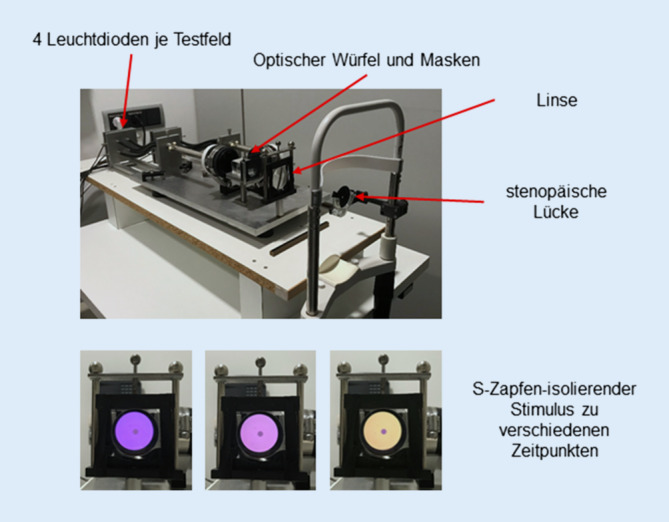

Der verwendete Leuchtdiodenstimulator ist in Abb. 3 dargestellt. Er besitzt 8 Leuchtdioden, deren Helligkeit mit hoher zeitlicher Auflösung unabhängig voneinander über eine Soundkarte gesteuert werden kann (je 2 rote: 660 nm, 2 grüne: 558 nm, 2 cyanfarbige: 516 nm und 2 blaue: 460 nm; Bandbreite der Spektren durch Interferenzfilter auf ~8 nm begrenzt). Die Lichtquellen werden über einen Maxwell-Strahlengang auf die Pupillarebene der Versuchspersonen projiziert, sodass hohe retinale Beleuchtungsstärken erzielt werden können. Das ringförmige Testfeld (Außendurchmesser 12° und Innendurchmesser 2°) wird im Strahlengang durch Prismen und Masken erzeugt, sodass es bei Fernakkomodation scharf auf die Netzhaut abgebildet wird.

Die verwendete Technik wurde in unseren früheren Publikationen bereits ausführlich beschrieben [9, 10, 12, 13]. Die spektrale Zusammensetzung und die Helligkeit des Testfeldes werden durch 4 Leuchtdioden gesteuert. Die retinale Beleuchtungsstärke variiert zyklisch, liegt aber im zeitlichen Mittel stets bei 294 Td. Die Farbe Testfeldes schwankt ebenfalls zyklisch um einen weißen Farbton (CIE-Koordinaten von x = 0,38 und y = 0,28). Das kreisförmige innere Testfeld (Durchmesser 2°) diente im Rahmen der hier vorgestellten Experimente lediglich als Fixationsziel (Helligkeit 147 Td, CIE-Koordinaten wie oben).

Die zyklischen Reize wurden in Endlosschleife präsentiert, bis die Versuchspersonen mittels Knopfdruck (ja/nein) Rückmeldung gaben, ob eine zeitliche Veränderung im Testfeld wahrgenommen wurde oder nicht. Wir ermutigten die Probanden nach ca. 2–10 s zu antworten. Zur Bestimmung der zeitlichen Kontrastschwelle wurde zunächst der maximale Kontrast (Treppe 1) oder kein Kontrast (Treppe 2) präsentiert und die Reizstärke abhängig von der Antwort mit fester Schrittgröße verringert oder erhöht, wobei die Schrittgröße bei jeder Veränderung der Antwort (ja zu nein oder nein zu ja) halbiert wurde. Die beiden Treppen wurden zufällig ineinander verschränkt.

Die Helligkeiten der Leuchtdioden wurden für unsere Experimente mit unterschiedlicher zeitlicher Frequenz sinusförmig um die oben genannten mittleren Einstellungen herum moduliert. Die Modulation erfolgte entweder in Phase (0°) oder in Gegenphase (180°). Dabei wurden Kontrast und Phase der Modulation mittels Matrizenrechnung auf der Basis der unterschiedlichen spektralen Empfindlichkeiten der Photorezeptortypen („cone fundamentals“) so berechnet, dass sich die Rate der Photoisomerisationen lediglich in einem Photorezeptortyp veränderte (Triple-Silent-Substitution) [7, 8, 24]. Die Modulation des Testfeldes kann also nur über diese Photorezeptorart wahrgenommen werden [15]. Die Kontraste auf der Ebene der Photorezeptoren können unter Beibehaltung der Isolation durch Veränderung der Kontraste aller 4 Leuchtdioden in gleichem Verhältnis variiert werden.

### Molekulargenetische Sicherung

Genomische DNA wurde aus Blutlymphozyten nach Standardprotokoll extrahiert. Die NGS-basierte molekulargenetische Diagnostik erfolgte mit auf den klinischen Phänotyp Makuladystrophie bzw. Retinitis pigmentosa konzipierten Genpanels (Makuladystrophie: 22 Gene; Retinitis pigmentosa: 96 Gene). Die Anreicherung der jeweiligen kodierenden und flankierenden Regionen der Gene erfolgte mit einem Agilent SureSelectXT^TM^ Kit und nachfolgender Sequenzierung auf der Illumina MiSeq Platform. NGS-Daten wurden mit dem CLC Genomics Server (Aarhus, Denmark) ausgewertet. Der ORF15 des *RPGR*-Gens wurde mit Sanger-Sequenzierung untersucht. Die Klassifizierung der Sequenzvarianten richtete sich nach den aktuellen Empfehlungen des ACMG und AMP.

## Ergebnisse

### Okkulte Makuladystrophie

Bei den beiden ersten Patienten (#1 und #2) zeigte sich eine deutliche Visusminderung mit regelrechtem fundoskopischem Befund. Im Gesichtsfeld bestand ein relatives Zentralskotom. Die Fundusautofluoreszenz war unauffällig, und im OCT fand sich eine Rarefizierung des zweiten reflektiven Bandes (entspricht dem Übergang von Innen- zu Außensegmenten der Photorezeptoren). Der morphologische Befund von Patientin #2 ist charakteristisch und in Abb. 1 dargestellt. In der Elektrophysiologie fand sich ein normales Blitz-ERG ohne Hinweis auf eine generalisierte Zapfendystrophie.

Der erste Patient (#1), 54 Jahre und männlich, wies einen bestkorrigierten Visus von rechts 0,1 und links 0,125 auf. Es bestand eine Myopie von −6 dpt. Im multifokalen ERG (Daten nicht gezeigt) fanden sich verminderte zentrale Antworten.

**Figure 4:**
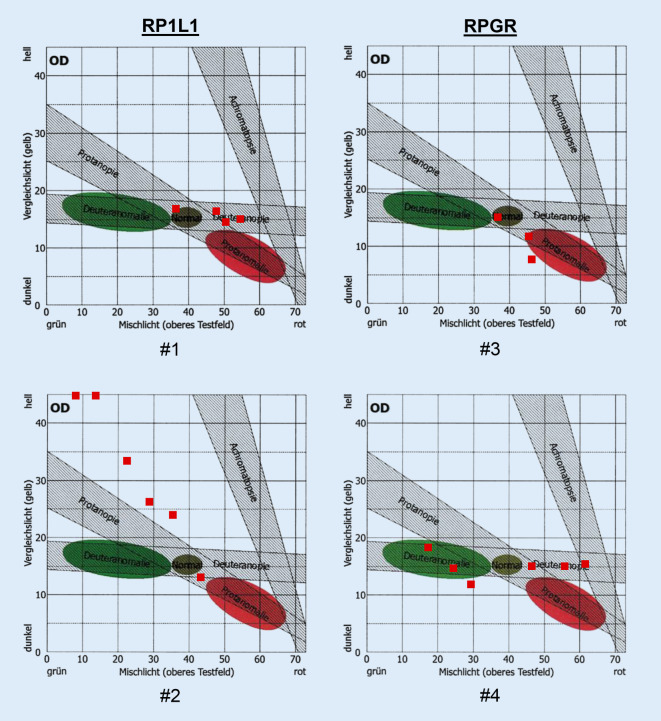

Die Einstellbreite in der Untersuchung mit dem Anomaloskop war nur mittelgradig vergrößert bei fehlender Skotopisation (Abb. 4). Bei sonst regelrechtem Befund könnte das Resultat der Untersuchung auch als Deuteranomalie eingeordnet werden. Der Cambridge-Color-Test war deutlich pathologisch und die photorezeptorspezifische Kontrastempfindlichkeit vermindert (Tab. 1).

**Table 1:** 

Parameter	#1: RP1L1	#2: RP1L1	#3: RPGR	#4: RPGR	Normal
Geschlecht	Männl.	Weibl.	Weibl.	Weibl.	–
Alter	54	67	47	29	–
Bestkorrigierter Visus	0,1	0,1	0,25	0,4	–
CCT: Protan	95,1	110+^a^	–	74,6	10
CCT: Deutan	94,5	110+^a^	–	85,2	10
CCT: Tritan	87,9	99,1	–	102,4	15
Anomaloskop: Einstellbreite	0,78	9,86	0,52	3,59	–
Anomaloskop: Mittelwert	0,81	5,57	0,96	2,04	1,0
Anomaloskop: Skotopisation	Nein	Ja	Nein	Nein	–
Log KS: L‑Zapfen 2 Hz	1,73	1,43	1,94	1,89	2,12
Log KS: M‑Zapfen 2 Hz	2,06	1,52	2,01	1,58	2,22
Log KS: S‑Zapfen 2 Hz	0,91	0,87	1,15	0,91	1,49

^a^Bei 110 ist der maximal darstellbare Kontrast erreicht – höhere Werte können also nicht gemessen werden

Bei der zweiten 67-jährigen Patientin (#2, Abb. 1a, c, e) bestand langjährig eine Visusminderung beidseits auf 0,1 (sphärisches Äquivalent 0,625 dpt). Der Fundusbefund war ebenfalls unauffällig, und die Patientin klagte nicht über eine ausgeprägte Blendempfindlichkeit. Das Anomaloskop zeigte eine ausgeprägte Farbsinnstörung mit einer vergrößerten Einstellbreite und einer deutlichen Reduzierung der Helligkeit bei zunehmenden Rotanteilen (Skotopisation). Auch der Cambridge-Color-Test wies ausgeprägte Verminderungen der Farbdiskrimination entlang aller 3 Achsen auf.

In der Zusammenschau der Befunde wurde bei beiden Patienten eine okkulte Makuladystrophie vermutet und genetisch gesichert, beide waren heterozygote Träger der dominant vererbten c.133C>T/p.(Arg45Trp) Mutation im *RP1L*-Gen.

### Heterozygote Konduktorinnen von RPGR-Mutationen

Die beiden heterozygoten *RPGR*-Konduktorinnen hatten jeweils eine einen verminderten Visus und eine mäßiggradige Gesichtsfeldeinschränkung. Der Fundusbefund zeigte einen veränderten Fundusreflex und vereinzelt periphere Pigmentierungen sowie ein reduziertes, aber nicht erloschenes Ganzfeld-ERG.

Eine 47-jährige Patientin (#3) hatte extern bereits die Diagnose einer Retinitis pigmentosa bekommen. Fundoskopisch lag bei ihr ebenfalls ein relativ unauffälliger Befund mit lediglich vereinzelten peripheren Pigmentierungen und einem mittelgradig konzentrisch eingeschränkten Gesichtsfeld vor. Das Ganzfeld-ERG war reduziert, aber nicht erloschen. Der bestkorrigierte Visus liegt bei 0,4 OD und 0,5 OS (sphärisches Äquivalent von −4,5 dpt). Die Patientin hat einen Sohn, welcher von der RP bereits zu diesem Zeitpunkt deutlich stärker betroffen war. Es fanden sich vergleichbar Einschränkungen im Farbsinntest wie bei der vorher genannten Patientin (#3). Bei der Patientin und ihrem Sohn wurde eine c.2405_2406delAG/p.(Glu802Glyfs*32)-Mutation im *RPGR*-Gen hetero- bzw. hemizygot nachgewiesen.

**Figure 5:**
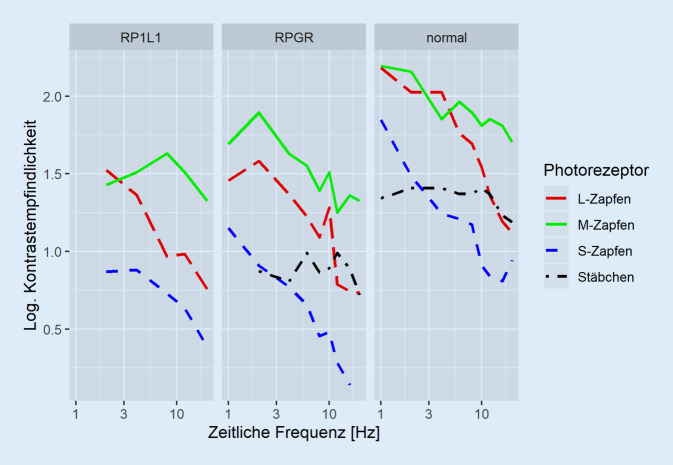

Eine 29-jährige Patientin (#4, Abb. 1b, d, f) wurde mit Verdacht auf Pigmentosa sine pigmento vorgestellt. Fundoskopisch war der zentrale Reflex vermindert, und es fanden sich einzelne pflastersteinartige Veränderungen in der Peripherie. Das Gesichtsfeld war mäßiggradig konzentrisch eingeschränkt und das Ganzfeld-ERG nach ISCEV-Standard skotopisch wie auch photopisch deutlich reduziert. Der Visus betrug beidseits 0,25 bei einer deutlichen Myopie (um −10 dpt). Auch hier zeigten sich ausgeprägte Auffälligkeiten im Farbsinn mit einer vergrößerten Einstellbreite im Anomaloskop ohne ausgeprägte Skotopisation und verminderter Farbdiskriminierung im CCT. Die photorezeptorspezifische zeitliche Kontrastempfindlichkeit war ebenfalls vermindert (Abb. 5). Bei der Patientin wurde eine c.247G>A/p.(Ala83Thr)-Mutation im *RPGR*-Gen heterozygot nachgewiesen.

Im OCT wiesen beide Patientinnen deutlich weniger ausgeprägte morphologische Veränderungen der äußeren Netzhaut im Vergleich zu den *RP1L1*-Patienten auf. Dafür lag ein pathognomonisches Fundusautofluoreszenzmuster vor.

### Vergleich der Farbsinntests zwischen den Gruppen

Eine vergrößerte Einstellbreite in der Rayleigh-Gleichung fand sich bei allen 4 Patienten, eine für die Zapfendystrophien und die Achromatopsie/Blauzapfenmonochromasie typische Skotopisation jedoch nur bei Patientin #2 (*RP1L1*). Eine Abgrenzung zu den X‑chromosomalen Farbsinnstörungen kann unter Umständen schwierig sein und bereitet insbesondere bei männlichen Patienten Schwierigkeiten. Im Cambridge-Color-Test fanden sich deutlich erhöhte Schwellen entlang aller 3 Farbachsen. Die vollständigen Kurven der zeitlichen Kontrastempfindlichkeit in Abhängigkeit von der Frequenz sind in Abb. 5 dargestellt. Es fanden sich bei beiden Patientinnen leicht verminderte Empfindlichkeiten für L‑ und M‑Zapfen-isolierende Reize. Bei der *RPGR*-Patientin wurden auch Stäbchen-isolierende Reize gemessen, welche ebenfalls reduziert waren.

## Diskussion

Die typische funktionelle Trias bei den Zapfendystrophien sind Visusminderung, Blendempfindlichkeit und Farbsinnstörungen. Farbsinnstörungen folgen nicht zwangsläufig den klassischen Verwechslungsachsen der X‑chromosomal-vererbten anomalen Trichromasien bzw. Dichromasien. Bei fortgeschrittenen Zapfendystrophien finden sich typischerweise Farbsinnstörungen Typ I nach Verriest mit Verwechslungen v. a. entlang der Protanachse und der sog. Skotopisation [26]. Bei der Skotopisation gewinnen die Stäbchen bei der Untersuchung mit dem Anomaloskop zunehmend Einfluss auf den Farbabgleich, was zu hohen Empfindlichkeiten im grünen und ausgeprägtem Abfallen der Empfindlichkeit im roten Bereich führt. Bei der Retinitis pigmentosa finden sich häufig weniger ausgeprägte Störungen, welche eher entlang der Blau-Gelb-Achse verlaufen, und gelegentlich auch eine Pseudoprotanomalie [11, 21, 25]. Allerdings kommen auch Störungen wie bei den Zapfendystrophien vor [11].

Ältere Beschreibungen von Farbsinnstörungen bei den relativ breit definierten und sehr heterogenen Erkrankungen Pigmentosa oder Zapfendystrophien erweisen sich mit wachsendem Wissen über die genetischen Grundlangen als zunehmend unzureichend. Zu den einzelnen genetischen Formen hingegen bestehen oft keine detaillierten Beschreibungen der Farbsinnstörungen, da die relativ komplizierten sinnesphysiologischen Untersuchungsmethoden aufgrund der Fortschritte in der Bildgebung zunehmend ins Hintertreffen geraten. Ob dies ein grundsätzliches, unüberwindliches Problem darstellt („Farbsinnstörungen sind zu unspezifisch“), oder ob der Nachweis deutlich besser definierter funktioneller Veränderungen des Farbsinnes in Zukunft einen diagnostischen Stellenwert haben könnte, ist Gegenstand unserer Forschung.

In der Praxis werden v. a. die pseudoisochromatischen Ishihara-Tafeln und der Farnsworth-Panel-D15-Test in der gesättigten oder ungesättigten Variante verwendet. Hierbei ist zu beachten, dass der gesättigte Panel-D15-Test keine sehr hohe Sensitivität aufweist und z. B. auch von Personen mit milder anomaler Trichromasie bestanden werden kann [2]. Das Anomaloskop mit der Rayleigh-Gleichung erlaubt die genaue Einordnung und auch eine gewisse Quantifizierung der Rot-Grün-Störung. Computerbasierte Verfahren wie der Cambridge-Color-Test und der Color-Assessment-and-Diagnosis-Test (CAD-Test) sind noch nicht so weit verbreitet, erlauben aber ebenfalls eine Einordnung und Quantifizierung der Farbsinnstörung, wobei sie das Anomaloskop nicht vollständig ersetzen können [11]. Nur das Anomaloskop erlaubt direkte Aussagen über die spektralen Eigenschaften der Photopigmente, da diese Untersuchungsmethode auf metamerem Abgleich beruht (d. h. auf der Einstellung von Farbmischungen mit identischer Aktivierung der Fotorezeptoren) und nicht auf Farbverwechslung oder -diskriminierung [11]. Die Messung der photorezeptorspezifischen Kontrastempfindlichkeit (DeLange-Kurven) ist noch nicht ausreichend etabliert und bleibt spezialisierten sinnesphysiologischen Labors vorbehalten [9, 10, 12].

Mutationen im *RP1L1*-Gen (Chromosom 8) führen zur autosomal-dominant vererbten okkulten Makuladystrophie [1] (gelegentlich auch zu einer RP [5]). Das klinische Bild ist durch Visusminderung bei regelrechter Fundoskopie und Fluoreszenzangiographie, verminderte Antworten in den zentralen Feldern des multifokalen Elektroretinogramms und Auffälligkeiten der äußeren Netzhautschichten im OCT gekennzeichnet [1, 14, 28]. Farbsinnstörungen sind vorhanden und wurden in einigen Studien untersucht [14, 20]. Es gibt auch Hinweise auf eine Störung der Stäbchenfunktion.

Im Gegensatz dazu sind Mutationen im *Retinitis-pigmentosa-GTPase-Regulator*-Gen zuerst im Zusammenhang mit der X‑chromosomalen Retinitis pigmentosa beschrieben worden [16, 18]. Sie können aber auch Zapfen-Stäbchen-Dystrophien auslösen [6]. Es wird davon ausgegangen, dass ca. 70 % der X‑chromosomalen RP-Formen durch Mutationen in diesem Gen verursacht sind. Betroffen ist das zentrale Cilium, welches am Übergang vom Innen- zum Außensegment der Photorezeptoren eine wesentliche Rolle für den intrazellulären Transport spielt [16, 18]. Symptome sind auch bei heterozygoten weiblichen „Konduktorinnen“ häufig vorhanden, sodass manche Autoren vorschlagen, von eine X‑chromosomal-dominanten Vererbung zu sprechen. Die Konduktorinnen weisen oft eine große Spannbreite an Schweregraden auf, welche von Auffälligkeiten im ERG und im Fundusreflex bei sonst symptomfreien Patientinnen bis hin zu vergleichbar schweren RP-Formen wie bei hemizygoten männlichen Patienten reichen [17]. Bei den von uns untersuchten Patientinnen fanden sich ein relativ unauffälliger Fundusbefund mit leichten Pigmentveränderung, Pflastersteinen und vermindertem Fundusreflex, eine milde konzentrische Gesichtsfeldeinschränkung, aber ein deutlicher Visusverlust und ausgeprägte Farbsinnstörungen. Diagnostisch wegweisend kann bei diesen Patientinnen neben der Familienanamnese v. a. das von Wegscheider, Preising und Lorenz erstmals beschriebene pathognomonische Autofluoreszenzmuster sein [27].

In unserer Studie fanden sich bei einer Patientin mit okkulter Makuladystrophie die ausgeprägtesten Farbsinnstörungen. Sie wies eine Skotopisation im Anomaloskop auf und zeigte die höchsten Schwellen im Cambridge-Color-Test. Die photorezeptorspezifische zeitliche Kontrastempfindlichkeit war hingegen nur mäßig vermindert. Der Grund hierfür ist vermutlich das relativ große Testfeld, welches einen optischen Winkel von 12° ausfüllt. Die Wahrnehmung könnte somit über die weiter peripher gelegenen, perifovealen Netzhautareale vermittelt werden, welche bei der okkulten Makuladystrophie weniger oder gar nicht beteiligt sind. Die räumlichen Verhältnisse sind übrigens auch bei den anderen Verfahren zur Messung des Farbsinns zu berücksichtigen. Dies gilt bei den erworbenen Farbsinnstörungen, wo die zugrunde liegende Pathologie ungleich über die Netzhaut verteilt sein kann, mehr als bei den X‑chromosomalen Farbsinnstörungen. So erscheint das Testfeld beim Anomaloskop unter einem Winkel von 2° und beim CCT unter einem Winkel von 7°. Zur Bestimmung der photorezeptorspezifischen zeitlichen Kontrastempfindlichkeit muss in Zukunft eine flexiblere räumliche Gestaltung bei den Techniken erreicht werden.

Insgesamt können neue Untersuchungsmethoden eine verbesserte funktionelle Charakterisierung des Phänotyps ermöglichen und damit in der Forschung wertvolle Dienste leisten. Darüber hinaus ist aber auch zu hoffen, dass sie ein klinisches Einsatzgebiet finden und ggf. helfen, Therapieeffekte in klinischen Studien zu demonstrieren.

## Fazit für die Praxis

- Das *RP1L1*-Gen (Chromosom 8) ist verantwortlich für die autosomal-dominante okkulte Makuladystrophie, das *RPGR*-Gen (X‑Chromosom) für die X‑chromosomale Pigmentosa.
- In beiden Fällen können heterozygote Träger eine deutlich gestörte Zapfenfunktion haben.
- Der fundoskopische Befund kann relativ unauffällig sein, es finden sich jedoch Auffälligkeiten im OCT oder in der FAF.
- Der Nachweis ausgeprägter Farbsinnstörungen kann bei der Diagnosestellung hilfreich sein.
- Neue Ansätze zur Quantifizierung der Farbsinnstörung bzw. der Zapfenfunktion könnten in Zukunft als Verlaufsparameter für klinische Studien geeignet sein.
